# Bioactivity Signatures of Drugs vs. Environmental Chemicals Revealed by Tox21 High-Throughput Screening Assays

**DOI:** 10.3389/fdata.2019.00050

**Published:** 2019-12-13

**Authors:** Deborah K. Ngan, Lin Ye, Leihong Wu, Menghang Xia, Anna Rossoshek, Anton Simeonov, Ruili Huang

**Affiliations:** ^1^Division of Pre-clinical Innovation, National Center for Advancing Translational Sciences (NCATS), National Institutes of Health (NIH), Rockville, MD, United States; ^2^National Center for Toxicological Research, US Food and Drug Administration, Jefferson, AR, United States

**Keywords:** *in vitro* assay, quantitative high throughput screening, Tox21, drug, environmental chemical

## Abstract

Humans are exposed to tens of thousands of chemicals over the course of a lifetime, yet there remains inadequate data on the potential harmful effects of these substances on human health. Using quantitative high-throughput screening (qHTS), we can test these compounds for potential toxicity in a more efficient and cost-effective way compared to traditional animal studies. Tox21 has developed a library of ~10,000 chemicals (Tox21 10K) comprising one-third approved and investigational drugs and two-thirds environmental chemicals. In this study, the Tox21 10K was screened in a qHTS format against a panel of 70 cell-based assays with 213 readouts covering a broad range of biological pathways. Activity profiles were compared with chemical structure to assess their ability to differentiate drugs from environmental chemicals, and structure was found to be a better predictor of which chemicals are likely to be drugs. Drugs and environmental chemicals were further analyzed for diversity in structure and biological response space and showed distinguishable, but not distinct, responses in the Tox21 assays. Inclusion of other targets and pathways to further diversify the biological response space covered by these assays is likely required to better evaluate the safety profile of drugs and environmental chemicals to prioritize for in-depth toxicological studies.

## Introduction

One of the major challenges in the commercial market is the accurate assessment of potential adverse effects of approved drugs and environmental chemicals on human health. All drugs available for sale in the U.S. have been approved by the U.S. Food and Drug Administration (FDA) to ensure that they work correctly and that their health benefits outweigh their known and potential risks (U.S. Fod Drug Administration, 2018). Similarly, environmental chemicals (ENVCs), defined by the Centers for Disease Control and Prevention (CDC) as “a chemical compound or chemical element present in air, water, food, soil, dust, or other environmental media such as consumer products,” (Centers for Disease Control Prevention, 2017) are required to be analyzed for safety by the Environmental Protection Agency (EPA) prior to releasing to the market (United States Environmental Protection Agency, 2017). As a result of the independent evaluation processes, different regulatory decisions may be required for the assessment of drugs and ENVCs. Marketed drugs may be considered unsafe after exhibiting unexpected toxicity in the clinic, and these drugs are often recalled for this very reason (Saluja et al., 2016). Furthermore, older drugs that were not subjected to the same rigorous FDA approval process and “grandfathered” into the market were generally not sufficiently tested for safety and effectiveness prior to human use (Chhabra et al., 2005; Nasr et al., 2011). As for ENVCs, there is a general lack of information on their potential toxicological effects (Huang et al., 2016). People are exposed to thousands of drugs and ENVCs during their lifetime, presenting the need for the development of high-throughput methods to systematically evaluate the toxicity of these chemicals. While filling in the toxicity data gap, such methods also need to be assessed to determine if they are suitable for characterizing both types of chemicals.

The Toxicology in the Twenty-First Century (Tox21) partnership (NRC, 2007; Collins et al., 2008; Kavlock et al., 2009; Tice et al., 2013) has developed *in vitro* methods with the aim to rapidly and efficiently evaluate the safety of commercial chemicals, pesticides, food additives/contaminants, and medical products (Shukla et al., 2010). In Tox21, quantitative high-throughput screening (qHTS) (Attene-Ramos et al., 2013, 2015; Hsu et al., 2014; Huang et al., 2014, 2018; Hsieh et al., 2015), an automated robotic process in which each compound of a large chemical library is tested at multiple concentrations, is used to test large collections of chemicals in a battery of cell-based assays (Huang et al., 2016). A collection of ~10,000 ENVCs, as well as approved and investigational drugs, called the Tox21 10K library, has been screened for potential biological pathway disruptions that may result in toxicity. The NCATS Pharmaceutical Collection (NPC), a collection of small molecule drugs approved for clinical use or investigational purposes, is also part of the Tox21 10K library (Huang et al., 2011a). This library has been tested against ~70 cell-based assays from the Tox21 testing pipeline in qHTS format and has generated nearly 100 million data points to date (Huang et al., 2016, 2018; NCATS, 2016). The assay panel focuses on two major biological areas: nuclear receptor signaling (Huang et al., 2011b) and stress response pathways (Shukla et al., 2010), and also includes a smaller number of assays that probe for genotoxicity (Witt et al., 2017), developmental toxicity, and cell-death signaling (Hsieh et al., 2017).

QSAR modeling has been widely used to predict the drug-likeness of a molecule, but these methods have focused mostly on screening for ADME-Tox properties (Clark and Pickett, 2000; Di and Kerns, 2016; Shin et al., 2016). The rich set of compound activity profiles established across a wide spectrum of Tox21 assays provided us with the unique opportunity to characterize the biological responses of drugs vs. ENVCs. Drugs are generally expected to be better characterized, more target specific, and thus exhibit response profiles different from those of ENVCs in a panel of toxicity-focused assays such as the Tox21 assays. The Tox21 assay panel, on the other hand, could capture unexpected drug side effects that result in toxicity. Ideally, an optimally designed panel of assays should be able to distinguish drug-like characteristics from ENVCs, and these different safety profiles could be applied to help decision-making on both prioritization for in-depth toxicological studies and drug development. The Tox21 10K library is comprised of approximately one-third drugs and two-thirds ENVCs (Huang et al., 2011a). To evaluate the suitability of the current Tox21 assay panel for assessing drug and ENVC toxicity, we compared the biological responses of all drugs and ENVCs in the Tox21 10K compound collection when screened against ~70 assays with 213 different readouts. In addition, computational models were built to explore if drugs in the 10K compound library could be identified using the *in vitro* assay activity profiles or chemical structure, i.e., if compound activity profiles, in comparison to chemical structure, could be used as signatures to predict which compounds are likely to be drugs. The data collectively gathered in this study can be applied to optimize the Tox21 assay panel which can lead to better characterization of drugs and ENVCs, improved *in vivo* toxicity prediction, and ultimately more effective prioritization of substances for additional investigational studies.

## Materials and Methods

### *In vitro* Assay and Structure Data

qHTS data generated from the Tox21 10K collection up to the end of 2018 were used for modeling, including 70 assays with 213 readouts (Supplementary Table 1) (Attene-Ramos et al., 2013; Huang et al., 2016, 2018). All data and detailed descriptions of these assays with target annotations are publicly available through the NCATS website (https://tripod.nih.gov/tox21/assays/) and PubChem (Wang et al., 2012; PubChem, 2016). Curve rank was used as the measure for compound activity (Huang et al., 2011b). The detailed process of data normalization, correction, classification of concentration response curves, and activity assignment was described previously (Huang, 2016). For modeling purposes, compounds with absolute curve rank >0.5 were set as active (1) and inactive (0) otherwise. ToxPrint chemotypes generated from the ChemoTyper (Yang et al., 2015) were used as fingerprints (729-bit) for structure-based models.

### Drug Prediction Modeling

In this study, only compounds that were included in the NPC subset library of the Tox21 10K collection were considered to be drugs. Any remaining drugs that were not part of the NPC library were removed from the ENVC category. A total of 8,427 unique compounds with structures available, including 3,739 drugs and 4,688 ENVCs, were used for chemical structure-based modeling. To obtain a balanced modeling set, a subset of ENVCs with a size roughly equal to the set of drug molecules was randomly chosen from the original dataset. The modeling set was randomly split into two sets, 70% for training and 30% for model validation, and this process was repeated 100 times. For assay activity-based models, compounds with complete profiles in the 70 assays (213 readouts), including 2,369 drugs and 4,735 ENVCs, were used for model training and validation. The same modeling procedure was repeated for the activity-based models. All compounds used for modeling were derived from the Tox21 10K collection, with ~80% of the compounds overlapping between the two sets used for structure- and activity-based models.

Models were built using the random forest (RF) method within R (version 3.5.0). RF is an ensemble learning method for classification and regression that operates by constructing multiple decision trees. Each tree is trained on roughly two-thirds of the total training data and gives a classification on the remaining one-third of data. The forest chooses the classification having the most votes over all the trees in the forest. For a binary dependent variable, the vote will be either YES or NO. The percent YES votes received is the predicted probability.

Model performance was evaluated by calculating the area under the receiver operating characteristic curve (AUC-ROC). The ROC curve is a graphical plot that illustrates the predictive ability of a binary classification model across different thresholds. The ROC curve is created by plotting true positive rates against false positive rates at various thresholds. The area under the curve (AUC) provides an aggregate measure of model performance. A larger AUC value indicates better classifier performance. A perfect predictive model would have an AUC of 1 and an AUC of 0.5 indicates a random classifier.

### Statistical Tests

Principal component analysis (PCA) was performed within R package version 3.5.0. PCA reduces a large number of independent variables into just a few variables (the principal components). These principal components are a linear combination of the original variables to project high dimensional data into low dimensional spaces (3D or 2D). In this study, the first two principal components, PC1 and PC2, were calculated based on the 28 physiochemical descriptors derived from KNIME® as well as the 213 assay readouts. Drugs and ENVCs were plotted using just the first two principal components to evaluate the distribution of these two groups of compounds in both chemical and bioassay space. Other statistical tests, such as the χ^2^-test, were performed in R package version 3.5.0.

## Results

### Summary of *in vitro* Assay Data Across All Test Substances

**Figure 1:**
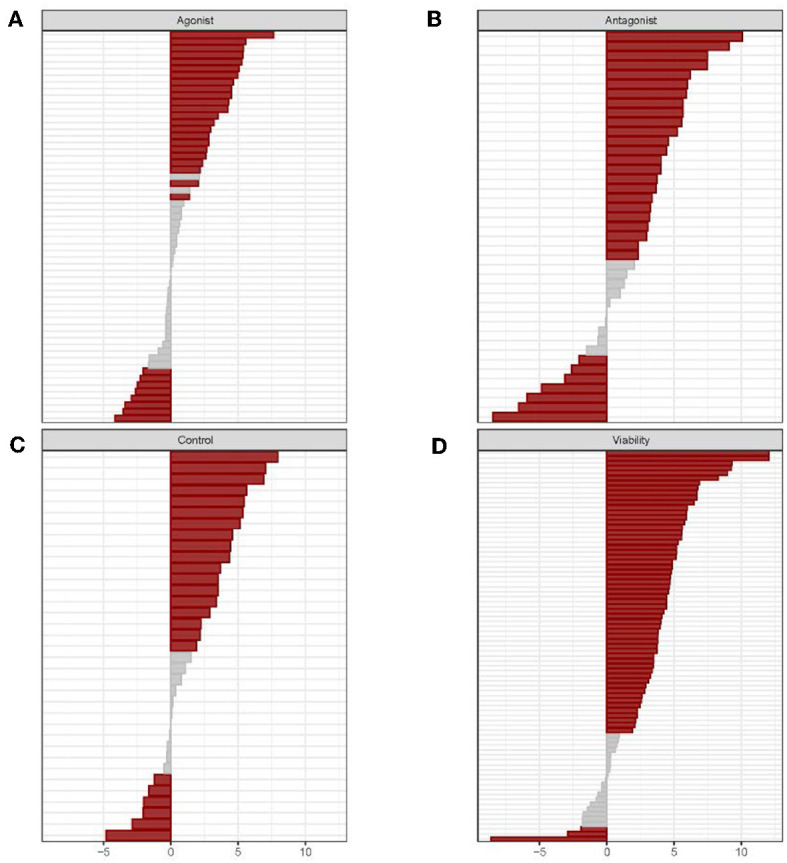
Individual assay responses of drugs and ENVCs grouped by readout type: **(A)** agonist, **(B)** antagonist, **(C)** control, and **(D)** viability. For each assay readout, the activities of drugs and ENVCs were compared using the χ^2^-test. Hit rate differences were determined by subtracting the ENVC hit rate from the drug hit rate. A *p*-value was calculated to determine the statistical significance of the hit rate difference. Assays marked in red signifies significant (*p* > 0.05) and gray otherwise.

High-throughput evaluations of the biological activity of drugs and ENVCs in the Tox21 10K compound library were conducted using 70 assays (213 readouts) from the Tox21 testing pipeline. The 213 readouts were divided up into four groups based on assay mode and readout type: agonist, antagonist, control, and viability. The agonist mode tested for compounds that induced the activity of a pathway/target, while the antagonist mode was used to identify compounds that inhibited the activity of a pathway/target. Furthermore, the control readout measured assay artifacts such as compound auto fluorescence and cytotoxicity. The control readout is not a counter screen, but rather it is one of the readouts generated from a multiple readout assay, such as the beta-lactamase (BLA) reporter gene assays (Huang et al., 2011b); not all assays have a control readout. Lastly, the viability readout assessed the integrity of the cells where a loss in signal indicates cell death or cytotoxicity. A summary of the assay activity of the drugs compared to ENVCs tested across the panel of Tox21 assays can be observed in Figure 1.

A χ^2^-test was performed to compare the hit rate (measured by curve rank; a compound with an absolute curve rank >0.5 was considered active or a hit) of the drugs and ENVCs in the Tox21 10K library in each assay readout and the statistical significance of the difference between the drug and ENVC hit rates were measured by a *p*-value from the χ^2^-test such that *p* < 0.05 was considered as statistically significant.

In Figure 1, the assays in each of the four readout types are sorted by increasing hit rate differences between drugs and ENVCs starting with highest value at the top and descending to the lowest value at the bottom. The gray regions located primarily in the middle of the graphs represent assays in which drugs and ENVCs did not show any significant difference in activity. The four readout types showed different distributions of significant assays, with “agonist” having the largest fraction of non-significant assays, followed by “viability,” “control,” and “antagonist.” The majority of assays in all four readouts revealed higher hit rates from drugs, thus indicating that drugs showed more activities than ENVCs.

Overall, ~68.5% of the assay readouts (146 out of 213 readouts) showed a significant difference (*p* < 0.05) between drug and ENVC responses. The assay readouts that showed the largest difference between drugs and ENVCs (smallest *p*-values) include (in descending significance): viability readout of ER-BG1 antagonist, viability readout of TR-beta antagonist, viability readout of AhR, viability of HepG2 cells at 0 h, and control readout of GR-BLA agonist, activity readout of TR-beta agonist, viability readout of FXR-BLA agonist, activity readout of GR-BLA agonist, viability readout of ER stress, and viability readout of PXR agonist. For the agonist readout, the assays with drugs showing more activities than ENVCs are: activity readout of GR-BLA agonist, activity readout of TR-beta agonist, activity readout of ERR agonist, activity readout of AR-BLA agonist, and activity readout of PPAR-gamma agonist. Likewise, the assays with more active ENVCs than drugs in this readout are: activity readout of CAR agonist, activity readout of ER-BG1 agonist (in the presence of antagonist), activity readout of ER-beta agonist, activity readout of hedgehog agonist, and activity readout of HSE-BLA. For the antagonist readout, the assays that exhibited the greatest drug activity compared to ENVCs are: activity readout of TR-beta antagonist, activity readout of ER-BLA antagonist, activity readout of hedgehog antagonist, activity readout of CAR antagonist, and activity readout of ER-BLA antagonist. ENVCs were more active in the following antagonist assay readouts: activity readout of RAR antagonist, activity readout of luciferase/biochemical, activity readout of mitochondria toxicity, activity readout of PGC-ERR, and activity readout of AR-BLA antagonist. For the viability readout, the assays that showed the highest activity among drugs include: ER-BG1 antagonist, TR-beta antagonist, FXR-BLA agonist, AhR, and Caspase-3/7 induction in CHO cells. Likewise, the assays for the viability readout that display the highest ENVC activity are: ER stress, SBE-BLA (TGF-beta) antagonist, mitochondria toxicity, RAR agonist, and RAR viability. A complete list of assay readouts and their significances in activity differences between drugs and ENVCs can be found in Supplementary Table 1.

### Hit Rate: Drugs vs. ENVCs

**Figure 2:**
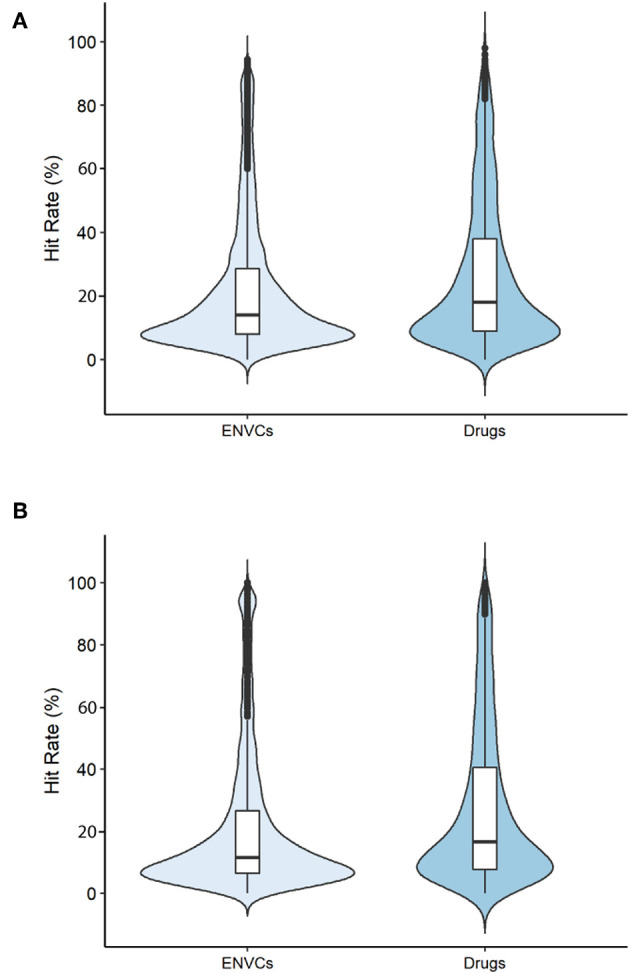
Hit rates of drugs and ENVCs when screened against **(A)** all assays or **(B)** only viability assays.

The hit rates of drugs and ENVCs were compared across two groups: all assays and only viability assays. The violin plots displayed in Figures 2A,B show the distribution of hit rates for drugs and ENVCs. These values were calculated by dividing the number of assay hits by the total number of assays tested.

When tested against all assays (Figure 2A), the median number of assays hit by ENVCs was ~14.1%, while the median value for drugs was 18.0%. Furthermore, when tested against only the viability assays (Figure 2B), the median percentage of assay hits for ENVCs and drugs were 11.4 and 18.5%, respectively. It is evident that both ENVCs and drugs showed similar hit rates when tested against all assays and only viability assays. The correlation in these values regardless of the type of assays they were tested against suggests that the two sets of compounds behaved similarly in the assays. It is important to note that ENVCs had a greater number of outliers than drugs, suggesting the ENVCs show a more diverse range of activities. When the compounds were tested against all assays, ~8.6% of ENVCs were outliers compared to only 3.0% of drugs. A similar trend was observed when compounds were tested against only the viability assays, with outliers comprising of 10.2% of ENVCs and 2.8% of drugs. On the other hand, the ENVCs showed a smaller interquartile range, across both all assays and viability assays only, when compared to drugs. Wider sections of the violin plot indicate a higher distribution of compounds sharing a particular hit rate, and narrower regions represent a lower distribution. The widest section of the violin plots when tested against all assays and viability assays only for both ENVCs and drugs fell within the range of 8–10% hit rate. Since drugs often have known targets for which they were designed, we would expect to see more on-target activity in drugs compared to ENVCs for certain biological assays. ENVCs, however, may have much more unintended biological activity depending on their use category, hence the need for further study on these substances to better understand the potential unintended toxic effects and harm they can cause to the human body.

The top five most active drugs when screened against all assays are tyrothricin, phenylmercuric borate, 4-chloromercuriphenol, alpha-tomatine, and auranofin; the most active ENVCs include basic blue 7, dichlone, rhodamine 6G, mercuric chloride, and trihexyltetradecylphosphonium bromide. A list of the top 30 active drugs and ENVCs and their hit rates when screened against all assays can be found in Tables 1A,B, respectively.

**Table 1:** Top 30 most active **(A)** drugs and **(B)** ENVCs from Tox21 10K library screens based on hit rates against all assays.

**#**	**CAS**	**Name**	**Hit rate (%)**
**(A)**			
1	1404-88-2	Tyrothricin	98.0
2	102-98-7	Phenylmercuric borate	98.0
3	623-07-4	4-Chloromercuriphenol	98.0
4	17406-45-0	alpha-Tomatine	96.0
5	34031-32-8	Auranofin	96.0
6	82318-06-7	Deslorelin acetate	96.0
7	298-83-9	Nitroblue tetrazolium dichloride	96.0
8	111358-88-4	Lestaurtinib	96.0
9	1055-55-6	Bunamidine hydrochloride	96.0
10	90-03-9	o-(Chloromercuri)phenol	96.0
11	114899-77-3	Trabectedin	96.0
12	218600-53-4	Bardoxolone methyl	96.0
13	548-62-9	Methylrosaniline chloride	95.8
14	458-37-7	Curcumin	94.3
15	65558-69-2	1,3-Diiminobenz(f)isoindoline	94.2
16	50935-04-1	Carminomycin	94.0
17	18378-89-7	Plicamycin	94.0
18	3380-34-5	Triclosan	92.5
19	2437-29-8	Malachite green oxalate	92.5
20	54965-24-1	Tamoxifen citrate	92.0
21	62-38-4	Phenylmercuric acetate	92.0
22	633-03-4	Brilliant Green	92.0
23	97-77-8	Disulfiram	91.5
24	97-18-7	Bithionol	91.1
25	68844-77-9	Astemizole	91.1
26	538-71-6	Domiphen bromide	91.1
27	140-66-9	4-tert-Octylphenol	90.6
28	70-30-4	Hexachlorophene	90.6
29	25155-18-4	Methylbenzethonium chloride	90.1
30	113-73-5	Gramicidin S	90.0
**(B)**			
1	2390-60-5	Basic Blue 7	94.4
2	117-80-6	Dichlone	93.4
3	989-38-8	Rhodamine 6G	92.5
4	7487-94-7	Mercuric chloride	91.5
5	654057-97-3	Trihexyltetradecylphosphonium bromide	91.5
6	23541-50-6	Daunorubicin Hydrochloride	91.5
7	137-30-4	Ziram	91.1
8	952-23-8	Proflavin hydrochloride	91.1
9	121107-18-4	Methyltrioctylammonium trifluoromethanesulfonate	91.1
10	1600-27-7	Mercury(II) acetate	90.6
11	1162-06-7	Triphenyllead acetate	90.6
12	14866-33-2	tetra-N-Octylammonium bromide	90.6
13	2772-45-4	2,4-Bis(1-methyl-1-phenylethyl)phenol	90.1
14	7173-51-5	Didecyldimethylammonium chloride	90.1
15	258864-54-9	Trihexyltetradecylphosphonium chloride	90.1
16	2390-68-3	Didecyldimethylammonium bromide	90.0
17	9004-95-9	Polyethylene Glycol Monocetyl Ether	90.0
18	79622-59-6	Fluazinam	89.7
19	13331-52-7	(Acryloyloxy)(tributyl)stannane	89.7
20	848641-69-0	1-Ethyl-3-methylimidazolium diethylphosphate	89.7
21	23906-97-0	Tetraoctylphosphonium bromide	89.7
22	701921-71-3	Trihexyltetradecylphosphonium dicyanamide	89.7
23	70862-65-6	1,3-Didecyl-2-methylimidazolium chloride	89.7
24	143-50-0	Chlordecone	89.2
25	76-87-9	Fentin hydroxide	89.2
26	2425-06-1	Captafol	89.2
27	1897-45-6	Chlorothalonil	89.2
28	100-56-1	Phenylmercury chloride	89.2
29	6317-18-6	Methylene dithiocyanate	89.2
30	5137-55-3	Methyltrioctylammonium chloride	89.2

*Hit rate is the percentage of assays in which the compound is active*.

When tested against only viability assays, many of the top active compounds overlapped with those screened against all assays. In fact, the top five most active drugs across all assays and viability assays are identical to each other in the same order of hit rate. The most active ENVCs in viability assays were similar to those across all assays, but there was more variability among ENVCs compared to drugs. The top five ENVCs in viability assays are didecyldimethylammonium bromide, polyethylene glycol (20) hexadecyl ether, tetrabutyltin, basic blue 7, and dichlone. Tables 2A,B presents a summary list of the top 30 active drugs and ENVCs in viability assays.

**Table 2:** Top 30 most active **(A)** drugs and **(B)** ENVCs from Tox21 10K library screens based on hit rates against only viability assays.

**#**	**CAS**	**Name**	**Hit rate (%)**
**(A)**			
1	1404-88-2	Tyrothricin	100
2	102-98-7	Phenylmercuric borate	100
3	623-07-4	4-Chloromercuriphenol	100
4	17406-45-0	alpha-Tomatine	100
5	34031-32-8	Auranofin	100
6	82318-06-7	Deslorelin acetate	100
7	1055-55-6	Bunamidine hydrochloride	100
8	90-03-9	o-(Chloromercuri)phenol	100
9	114899-77-3	Trabectedin	100
10	458-37-7	Curcumin	100
11	113-73-5	Gramicidin S	100
12	521-35-7	Cannabinol	100
13	25999-20-6	Lasalocid sodium	100
14	8004-87-3	Methyl Violet	100
15	2748-88-1	Miripirium chloride	100
16	2437-29-8	Malachite green oxalate	98.7
17	97-18-7	Bithionol	98.7
18	3380-34-5	Triclosan	97.5
19	54965-24-1	Tamoxifen citrate	97.5
20	70-30-4	Hexachlorophene	97.5
21	25155-18-4	Methylbenzethonium chloride	97.5
22	55-56-1	Chlorhexidine	97.5
23	1715-30-6	Alexidine dihydrochloride	97.5
24	58-27-5	Menadione	97.5
25	54767-75-8	Suloctdil	97.5
26	62-38-4	Phenylmercuric acetate	96.2
27	23593-75-1	Clotrimazole	96.2
28	538-71-6	Domiphen bromide	96.2
29	124-03-8	Ethylhexadecyldimethylammonium bromide	96.2
30	121-54-0	Benzethonium chloride	96.2
**(B)**			
1	2390-68-3	Didecyldimethylammonium bromide	100
2	9004-95-9	Polyethylene glycol (20) hexadecyl ether	100
3	1461-25-2	Tetrabutyltin	98.7
4	2390-60-5	Basic Blue 7	97.5
5	117-80-6	Dichlone	97.5
6	989-38-8	Rhodamine 6G	97.5
7	7487-94-7	Mercuric chloride	97.5
8	1600-27-7	Mercury(II) acetate	97.5
9	1162-06-7	Triphenyllead acetate	97.5
10	14866-33-2	Tetra-N-octylammonium bromide	97.5
11	23906-97-0	Tetraoctylphosphonium bromide	97.5
12	506-61-6	Potassium silver cyanide	97.5
13	130-61-0	Thioridazine hydrochloride	97.5
14	7774-29-0	Mercury(II) iodide	97.5
15	11024-24-1	Digitonin	97.5
16	101-96-2	N,N'-Bis(1-methylpropyl)-1,4-benzenediamine	97.5
17	3697-42-5	Chlorhexidine dihydrochloride	97.5
18	654057-97-3	Trihexyltetradecylphosphonium bromide	96.2
19	23541-50-6	Daunomycin hydrochloride	96.2
20	952-23-8	Proflavin hydrochloride	96.2
21	121107-18-4	Methyltrioctylammonium trifluoromethanesulfonate	96.2
22	13331-52-7	(Acryloyloxy)(tributyl)stannane	96.2
23	5137-55-3	Methyltrioctylammonium chloride	96.2
24	1166-52-5	Dodecyl gallate	96.2
25	900-95-8	Triphenyltin acetate	96.2
26	3282-73-3	Didodecyldimethylammonium bromide	96.2
27	4342-36-3	Tributyltin benzoate	96.2
28	171058-21-2	1-Methyl-3-tetradecylimidazolium chloride	96.2
29	203942-49-8	UK-337312	96.2
30	10540-29-1	Tamoxifen	96.2

*Hit rate is the percentage of assays in which the compound is active*.

While the top five most active ENVCs from the two tables are not exactly the same, there are many repeated compounds. Compounds that overlap between Tables 1, 2, such as the drugs tyrothricin, trabectedin, and curcumin and the ENVCs basic blue 7, rhodamine 6G, and mercury(II) acetate, indicate that their activity is most likely due to cytotoxicity or other assay artifacts such as compound auto fluorescence, as captured by the control readout. On the other hand, compounds that appear in Table 1 but not Table 2, such as the drugs lestaurtinib, bardoxolone methyl, and carminomycin and the ENVCs ziram, 2,4-bis(1-methyl-1-phenylethyl)phenol, and fluazinam, suggests that the unexpected activity of these compounds could be due to non-specific activities other than cytotoxicity (Simeonov et al., 2008; Jadhav et al., 2010).

### Comparison of Activity Profiles of ENVCs and Drugs by Category

**Figure 3:**
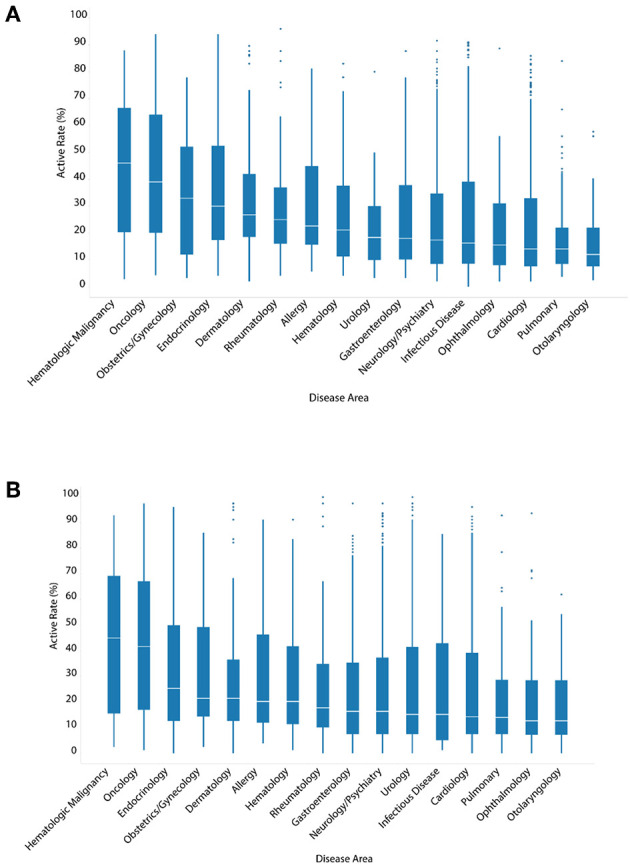
Hit rates of drugs by disease area when screened against **(A)** all assays, or **(B)** only viability assays.

Unlike ENVCs, most drugs for clinical use have known molecular targets. To examine the relationship between the hit rate of a drug and its intended clinical use, drugs were assigned at least one of 16 categories, when applicable, based on their specific disease area: hematologic malignancy, oncology, endocrinology, obstetrics/gynecology, dermatology, allergy, hematology, rheumatology, gastroenterology, neurology/psychiatry, urology, infectious disease, cardiology, pulmonary, ophthalmology, and otolaryngology (Figures 3A,B).

The hit rate distributions for each disease area are presented in a series of box-and-whisker plots. The oncology drugs showed the highest median number of assay hits: 39.0% of all assays and 41.8% of viability assays. This was expected because oncology drugs are often toxic chemicals that can disrupt normal cell function, such as replication and growth, resulting in cytotoxicity. The apparent high hit rates of these drugs against viability assays as well as all assays are likely a reflection of generic cytotoxic responses. Compared to oncology drugs, other disease areas, such as pulmonary and otolaryngology, were noticeably less active with median hit rates of 12.0–14.0%. The three disease areas with the highest drug counts were neurology/psychiatry, infectious disease, and cardiology. The median hit rates for these three disease areas in viability assays were 16.5, 15.2, and 14.3%, respectively. These values are comparable to the overall median hit rate (16.5%) of all drugs when tested against only viability assays. Figure 3B shows the hit rates of different drug categories in viability assays only to see how much of the drug activities observed could be attributed to cytotoxicity. With the exception of obstetrics/gynecology and rheumatology drugs, the cytotoxicity of the drugs in their respective disease areas correlated with their activity when tested against all assays.

**Figure 4:**
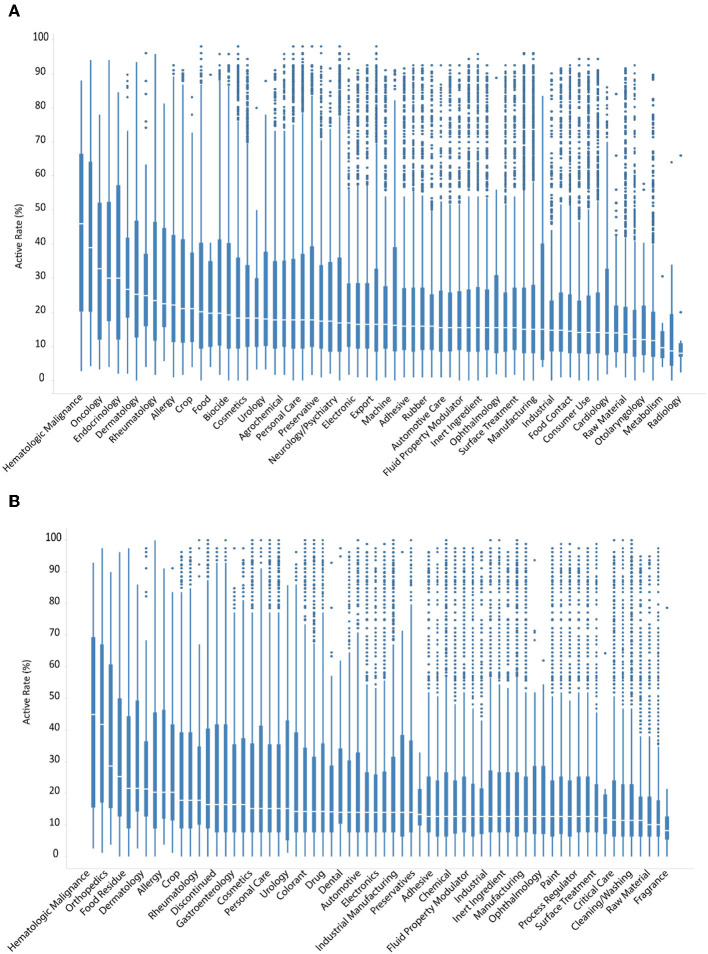
Hit rates of drugs and ENVCs by use category when screened against **(A)** all assays, or **(B)** only viability assays.

Contrary to clinically used drugs, ENVCs generally do not have as well-known or established cellular targets. Therefore, it is likely that these chemicals behave more unpredictably in various assays due to their less-studied nature. The compounds in the Tox21 10K library were grouped by different use categories, such as drug, manufacturing, pesticide, industrial manufacturing, consumer use, food additive, personal care, raw material, cosmetics, fragrance, and flavor. The full list of ENVCs use categories can be found on the EPA CompTox Chemicals Dashboard (Williams et al., 2017; EPA, 2018). These chemicals are presented as cumulative box-and-whisker graphs, with the most active category on the left and descending to the least active on the right (Figures 4A,B).

The categories with the highest hit rates include hematologic malignance, oncology, and dermatology, which are all drug categories (Figure 4A). The most active ENVCs categories included food residue, allergy, crop, drinking water contaminant, and food with median hit rates of 21.5, 20.3, 17.7, 17.7, and 16.5%, respectively (Figure 4B). The percentage of assay hits leveled out at ~14.0–18.0% for most of the remaining compounds. The ENVCs categories with the lowest hit rates include fragrance, flavor, cleaning/washing, food additive, and surface treatment with median hit rates of 10.1, 10.1, 11.4, 11.5, and 12.7%, respectively. Contrary to drugs, ENVCs had drastically more outliers in terms of both activity and cytotoxicity. This difference can mostly likely be attributed to the fact that ENVCs are not designed to hit any specific biological target and their activity would be much less predictable in these assays than drugs.

### Drug Prediction—Assay Activity vs. Chemical Structure

**Figure 5:**
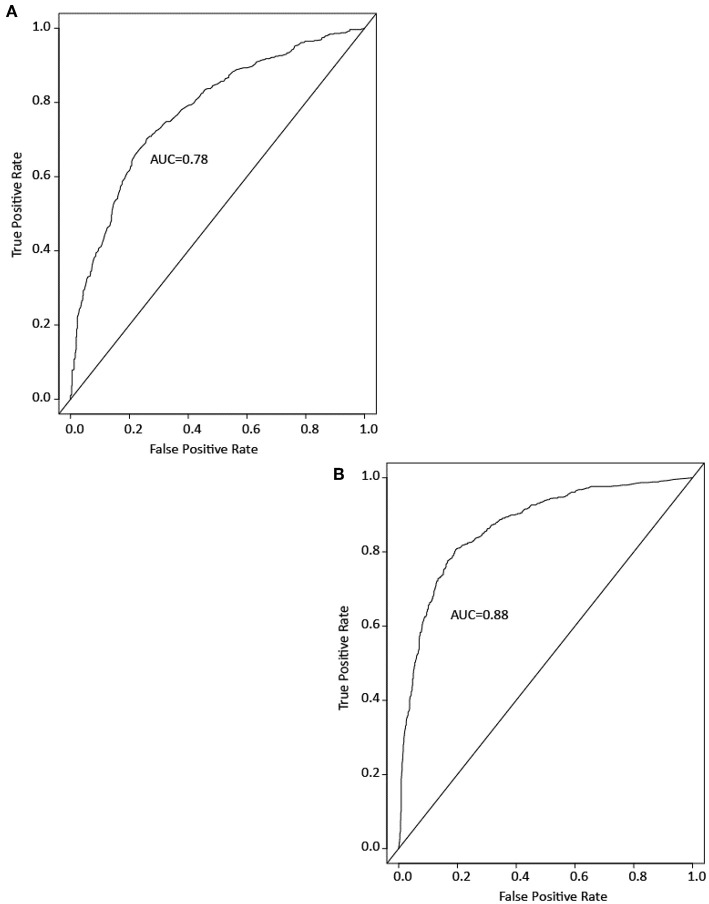
Example ROC curves of drug prediction models based on **(A)** assay activity and **(B)** chemical structure. Model performance is measured by the area under the ROC curve (AUC).

To see if drugs and ENVCs can be differentiated by their assay activity profile, we developed RF models to predict which compounds in the Tox21 10K library are likely to be drugs. Models were built using either the Tox21 assay data or chemical structure data. Model performance was measured by the area under the ROC curve (AUC). A good model performance would indicate that the drugs and ENVCs in the Tox21 library are distinguishable by activity or structure. Figure 5 shows example ROC curves from the models.

Both types of models performed well with an average AUC of 0.88 ± 0.01 for the structure-based model and 0.78 ± 0.01 for the assay activity-based model. These results indicate that the activity profiles of drugs in the Tox21 assays are sufficiently different from those of ENVCs, and these profiles can be used to identify drug-like compounds. However, the AUC of the activity-based model was lower than that of the structure-based model, suggesting that chemical structure is a better predictor of drugs.

The RF models also identified the structure features and assay readouts that contributed the most to differentiating drugs from ENVCs. The top five contributing structure features (in order of significance) are: aliphatic amine bond, heterocyclic ring, aromatic benzene ring, carboxamide bond, and amino carboxyl bond, which are common features found among drugs. As for assays, the top five contributing assay readouts are: activity readout of RAR antagonist, viability readout of PPAR-gamma antagonist, viability readout of TR-beta antagonist, activity readout of mitochondria toxicity, and viability readout of ER-BG1 antagonist. The complete list of significant chemical features and Tox21 assays can be found in Supplementary Figures 1, 2, respectively.

### Chemical and Biological Space Coverage—Drugs vs. ENVCs

**Figure 6:**
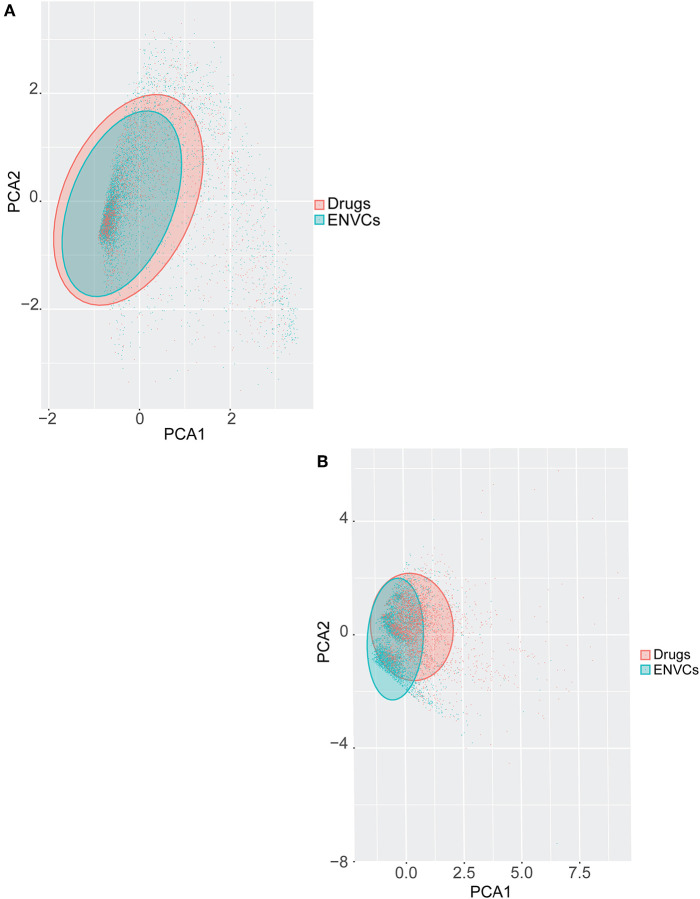
PCA of drugs and ENVCs using **(A)** Tox21 assay activity profiles, and **(B)** chemical structure data. The shaded area captures 95% of variance in data.

Principal component analysis (PCA) was used to visually represent the spatial distribution of both drugs and ENVCs (Figures 6A,B). As noted in the previous section, chemical structure performed better at predicting a compound's drug-likeness in the Tox21 10K collection than assay activity. Figure 6A shows the separation of drugs and ENVCs based on assay activity, where clusters of drugs are marked in red and ENVCs in teal. The variance of assay activity captured by the first two principle components are PC1: 48.8% and PC2: 3.3%. The separation of drugs and ENVCs based on chemical structure is illustrated in Figure 6B. In the shaded regions, clusters of red points representing drugs were shifted right in the spatial distribution while clusters of ENVCs noted by teal dots were shifted left. For the structure-based PCA using physiochemical descriptors, the variance captured by the first two principle components are PC1: 51.4% and PC2: 13.4%. The drug cluster suggests that they are structurally different from ENVCs and can generally be distinguished based on chemical structure. The better separation of drugs and ENVCs by structure compared to assay data is consistent with the modeling results, where chemical structure showed better performance, i.e., higher AUC, in predicting which compounds are likely to be drugs.

In Figures 6A,B, a teal shaded area representing ENVCs can be seen overlaying a red area marked for drugs. The shaded regions captured 95% of the variance in assay activity (Figure 6A) or chemical structure types (Figure 6B) based on the PCA. The larger shaded areas from drugs reveals that drugs covered a wider activity and structure space than ENVCs. The diverse activity observed in drugs matches the larger interquartile range seen in the box-plot distribution in Figure 2A. This is not surprising because drugs are designed to hit a diverse range of biological targets for different therapeutic purposes. In addition, to further compare the structures of drugs and ENVCs, we calculated the average nearest neighbor Tanimoto similarities of drugs to ENVCs, drugs among themselves, and ENVC among themselves. Both drugs (Tanimoto score: 0.748) and ENVCs (Tanimoto score: 0.827) were found to be more similar among themselves than to each other (Tanimoto score: 0.627), and the drugs appear to be more structurally diverse than ENVCs with a lower average nearest neighbor Tanimoto score. This is consistent with our PCA results where drugs covered a larger structural space than ENVCs.

## Discussion

The lack of a comprehensive understanding on the potential toxic effects of drugs and ENVCs in humans has revealed a grave need and urgency to better investigate the activity profiles of these compounds. Drugs, while diverse in their intended targeted areas, continue to present toxic effects that are often not detected in early pre-clinical and clinical studies. Likewise, ENVCs, while widely present in our everyday lives, suffer from a similar lack of knowledge about their potential toxicities. In this study, the Tox21 10K compound collection was screened against ~70 assays to test their biological responses. Drugs displayed greater hit rates when screened against the assay panel compared to ENVCs. We hypothesize that the difference in assay hits could be a result of drugs having more known biological targets than ENVCs and the fact that the present set of Tox21 assays covered mostly easy-to-test popular target classes and pathways with a wealth of known modulators. Drugs are intended to treat specified disease related biological conditions while most ENVCs are not intended to serve this same purpose.

The group of ENVCs included a notably higher number of outliers than drugs in terms of hit rates, some with very high hit rates. Since ENVCs do not undergo the same rigorous evaluation as drugs, they are likely to show more unexpected activity against different biological targets. These results reiterate the need to more thoroughly investigate the toxic effects of ENVCs prior to making them available on the commercial market. It was surprising to observe that some of the ENVC use categories were not as active as what one would expect. For instance, the median hit rate of pesticides was 15.2% of viability assays, which is lower than one might anticipate considering that pesticides are designed to kill organisms. The toxic effects of ENVCs on human health, including pesticides, is an important area of concern. Interestingly, in this study, the median percentage of actives from the collection of ENVCs tested was only 11.4%, whereas oncology drugs showed the highest median hit rate of 41.8% among viability assays, nearly four times the rate of ENVCs. This is not surprising since oncology drugs are generally designed to be cytotoxic, though the much lower pesticide hit rate underscores the need to screen a more diverse set of assays to fully evaluate the toxicological effects of ENVCs.

To further investigate if drugs and ENVCs showed distinct activity profiles in the Tox21 assays, we built computational models to see if the Tox21 assay data could be used to predict compounds in the Tox21 10K collection that are likely to be drugs. The assay activity-based models were further compared with models built with chemical structure data. The results showed that with the current set of assays, chemical structure was a better feature than assay activity in distinguishing drugs from ENVCs, given that the activity-based models also achieved good predictive performance. This may be due to the narrow biological space covered by the assays tested—with a heavy focus on nuclear receptors and stress response pathways—and in turn leads to the question of whether the current set of Tox21 assays included a sufficient variety of targets and pathways to comprehensively evaluate the activity, as well as toxicity, of drugs and ENVCs. Had a more diverse panel of assays been screened against these compounds, we could have better represented a comprehensive set of biological responses and gathered more accurate conclusions on the similarities and differences of the compound responses in the Tox21 collection, consistent with a previous analysis indicating that expanding the biological space coverage of the Tox21 assays, with G-protein-coupled receptor signaling and cytochrome P450 assays, for example, would lead to better prediction of *in vivo* drug toxicity (Huang et al., 2018). While RF modeling seemed to achieve modest success in distinguishing drugs from ENVCs based on either chemical structure or bioassay activity, the PCA results were not able to distinguish drugs from ENVCs when considering solely Tox21 activity data. The higher AUC obtained from the structure-based models indicated that chemical structures among various drugs have enough similarities to be distinct from those of the ENVCs.

Taken together, our results show that the drugs and ENVCs in the Tox21 10K library are distinguishable both by chemical structure and assay activity profile. Activity profile could be applied to predict which compounds are likely to be drugs, though not as precise a predictor as chemical structure, with the current set of assays. Drugs overall were consistently more active than ENVCs. Drugs also appeared to be more diverse, in terms of both assay activity and structure space coverage, when compared to ENVCs. The addition of new assays that cover biological space unexplored in this study could lead to better characterization of drug and ENVC activity profiles that could aid in the prioritization of compounds for in depth toxicological evaluation.

## Data Availability Statement

Publicly available datasets were analyzed in this study. This data can be found here: https://tripod.nih.gov/tox21/assays/.

## Author Contributions

DN and RH analyzed the data and wrote the manuscript. LY developed computational models and helped to write the manuscript. LW performed statistical analyses of the data. RH designed the analyses. RH, MX, AR, and AS managed the project. All authors reviewed the manuscript.

### Conflict of Interest

The authors declare that the research was conducted in the absence of any commercial or financial relationships that could be construed as a potential conflict of interest. The reviewer AS declared a past co-authorship with several of the authors RH and MX to the handling editor.

## References

[B1] Attene-Ramos M. S. Huang R. Michael S. Witt K. L. Richard A. Tice R. R.. (2015). Profiling of the Tox21 chemical collection for mitochondrial function to identify compounds that acutely decrease mitochondrial membrane potential. Environ. Health Perspect. 123, 49–56. 10.1289/ehp.1408642 25302578 PMC4286281

[B2] Attene-Ramos M. S. Miller N. Huang R. Michael S. Itkin M. Kavlock R. J.. (2013). The Tox21 robotic platform for the assessment of environmental chemicals–from vision to reality. Drug Discov. Today 18, 716–723. 10.1016/j.drudis.2013.05.015 23732176 PMC3771082

[B3] Centers for Disease Control Prevention (2017). Environmental Chemicals. Available online at: https://www.cdc.gov/biomonitoring/environmental_chemicals.html (accessed January 03, 2019).

[B4] Chhabra R. Kremzner M. E. Kiliany B. J. (2005). FDA policy on unapproved drug products: past, present, and future. Ann. Pharmacother. 39, 1260–1264. 10.1345/aph.1E569 15956239

[B5] Clark D. E. Pickett S. D. (2000). Computational methods for the prediction of 'drug-likeness'. Drug Discov. Today 5, 49–58. 10.1016/s1359-6446(99)01451-8 10652455

[B6] Collins F. S. Gray G. M. Bucher J. R. (2008). Toxicology. Transforming environmental health protection. Science 319, 906–907. 10.1126/science.1154619 18276874 PMC2679521

[B7] Di L. Kerns E. H. (2016). Drug-Like Properties - Concepts, Structure Design and Methods from ADME to Toxicity Optimization, 2 Edn. Burlington, MA: Academic Press.

[B8] EPA (2018). CompTox Chemicals Dashboard. Available online at: ftp://newftp.epa.gov/COMPTOX/Sustainable_Chemistry_Data/Chemistry_Dashboard/CPDat (accessed July 11, 2019).

[B9] Hsieh J. H. Huang R. Lin J. A. Sedykh A. Zhao J. Tice R. R.. (2017). Real-time cell toxicity profiling of Tox21 10K compounds reveals cytotoxicity dependent toxicity pathway linkage. PLoS ONE 12:e0177902. 10.1371/journal.pone.0177902 28531190 PMC5439695

[B10] Hsieh J. H. Sedykh A. Huang R. Xia M. Tice R. R. (2015). A data analysis pipeline accounting for artifacts in Tox21 quantitative high-throughput screening assays. J. Biomol. Screen. 20, 887–897. 10.1177/1087057115581317 25904095 PMC4568956

[B11] Hsu C. W. Zhao J. Huang R. Hsieh J. H. Hamm J. Chang X.. (2014). Quantitative high-throughput profiling of environmental chemicals and drugs that modulate farnesoid X receptor. Sci. Rep. 4:6437. 10.1038/srep06437 25257666 PMC4894417

[B12] Huang R. (2016). Chapter 12: A quantitative high-throughput screening data analysis pipeline for activity profiling, in High-Throughput Screening Assays in Toxicology, Vol. 1473, Methods in Molecular Biology, eds Zhu H. Xia M. (New York, NY: Humana Press), 111–122. 10.1007/978-1-4939-6346-1_12 27518629

[B13] Huang R. Sakamuru S. Martin M. T. Reif D. M. Judson R. S. Houck K. A.. (2014). Profiling of the Tox21 10K compound library for agonists and antagonists of the estrogen receptor alpha signaling pathway. Sci. Rep. 4:5664. 10.1038/srep05664 25012808 PMC4092345

[B14] Huang R. Southall N. Wang Y. Yasgar A. Shinn P. Jadhav A.. (2011a). The NCGC pharmaceutical collection: a comprehensive resource of clinically approved drugs enabling repurposing and chemical genomics. Sci. Transl. Med. 3:80ps16. 10.1126/scitranslmed.3001862 21525397 PMC3098042

[B15] Huang R. Xia M. Cho M. H. Sakamuru S. Shinn P. Houck K. A.. (2011b). Chemical genomics profiling of environmental chemical modulation of human nuclear receptors. Environ. Health Perspect. 119, 1142–1148. 10.1289/ehp.1002952 21543282 PMC3237348

[B16] Huang R. Xia M. Sakamuru S. Zhao J. Lynch C. Zhao T.. (2018). Expanding biological space coverage enhances the prediction of drug adverse effects in human using *in vitro* activity profiles. Sci. Rep. 8:3783. 10.1038/s41598-018-22046-w 29491351 PMC5830476

[B17] Huang R. Xia M. Sakamuru S. Zhao J. Shahane S. A. Attene-Ramos M.. (2016). Modelling the Tox21 10 K chemical profiles for *in vivo* toxicity prediction and mechanism characterization. Nat. Commun. 7:10425. 10.1038/ncomms10425 26811972 PMC4777217

[B18] Jadhav A. Ferreira R. S. Klumpp C. Mott B. T. Austin C. P. Inglese J.. (2010). Quantitative analyses of aggregation, autofluorescence, and reactivity artifacts in a screen for inhibitors of a thiol protease. J. Med. Chem. 53, 37–51. 10.1021/jm901070c 19908840 PMC2992957

[B19] Kavlock R. J. Austin C. P. Tice R. R. (2009). Toxicity testing in the 21st century: implications for human health risk assessment. Risk Anal. 29, 485–487; discussion: 492–487. 10.1111/j.1539-6924.2008.01168.x 19076321 PMC3202604

[B20] Nasr A. Lauterio T. J. Davis M. W. (2011). Unapproved drugs in the United States and the Food and Drug Administration. Adv. Ther. 28, 842–856. 10.1007/s12325-011-0059-4 21894470

[B21] NCATS (2016). Tox21 Data Browser. Available online at: https://tripod.nih.gov/tox21/ (accessed January 03, 2019).

[B22] NRC (2007). NRC Toxicity Testing in the 21st Century: A Vision and a Strategy. Washington, DC.

[B23] PubChem (2016). Tox21 Phase II Data. Available online at: http://www.ncbi.nlm.nih.gov/pcassay?term=tox21 (accessed November 04, 2019).

[B24] Saluja S. Woolhandler S. Himmelstein D. U. Bor D. McCormick D. (2016). Unsafe drugs were prescribed more than one hundred million times in the United States before being recalled. Int. J. Health Serv. 46, 523–530. 10.1177/0020731416654662 27302930

[B25] Shin H. K. Kang Y.-M. No K. T. (2016). Predicting ADME properties of chemicals. Handbook of Computational Chemistry, ed Leszczynski J. (New York, NY: Springer, 2265–2301.

[B26] Shukla S. J. Huang R. Austin C. P. Xia M. (2010). The future of toxicity testing: a focus on in vitro methods using a quantitative high-throughput screening platform. Drug Discov. Today 15, 997–1007. 10.1016/j.drudis.2010.07.007 20708096 PMC2994991

[B27] Simeonov A. Jadhav A. Thomas C. J. Wang Y. Huang R. Southall N. T.. (2008). Fluorescence spectroscopic profiling of compound libraries. J. Med. Chem. 51, 2363–2371. 10.1021/jm701301m 18363325

[B28] Tice R. R. Austin C. P. Kavlock R. J. Bucher J. R. (2013). Improving the human hazard characterization of chemicals: a Tox21 update. Environ. Health Perspect. 121, 756–765. 10.1289/ehp.1205784 23603828 PMC3701992

[B29] U.S. Fod Drug Administration (2018). Development & Approval Process (Drugs). Available online at: https://www.fda.gov/Drugs/DevelopmentApprovalProcess/default.htm (accessed January 04, 2019).

[B30] United States Environmental Protection Agency (2017). How EPA Evaluates the Safety of Existing Chemicals. Available online at: https://www.epa.gov/assessing-and-managing-chemicals-under-tsca/how-epa-evaluates-safety-existing-chemicals (accessed January 04, 2019).

[B31] Wang Y. Xiao J. Suzek T. O. Zhang J. Wang J. Zhou Z.. (2012). PubChem's bioassay database. Nucleic Acids Res. 40, D400–D412. 10.1093/nar/gkr1132 22140110 PMC3245056

[B32] Williams A. J. Grulke C. M. Edwards J. McEachran A. D. Mansouri K. Baker N. C.. (2017). The CompTox Chemistry Dashboard: a community data resource for environmental chemistry. J. Chem. Inform. 9:61. 10.1186/s13321-017-0247-6 29185060 PMC5705535

[B33] Witt K. L. Hsieh J. H. Smith-Roe S. L. Xia M. Huang R. Zhao J.. (2017). Assessment of the DNA damaging potential of environmental chemicals using a quantitative high-throughput screening approach to measure p53 activation. Environ. Mol. Mutagenesis 58, 494–507. 10.1002/em.22112 28714573 PMC5555817

[B34] Yang C. Tarkhov A. Marusczyk J. Bienfait B. Gasteiger J. Kleinoeder T.. (2015). New publicly available chemical query language, CSRML, to support chemotype representations for application to data mining and modeling. J. Chem. Inform. Model. 55, 510–528. 10.1021/ci500667v 25647539

