# Supplementary material for: Bioactivity Signatures of Drugs vs. Environmental Chemicals Revealed by Tox21 High-Throughput Screening Assays
Source: Front Big Data. 2019 Dec 13;2:50. doi: 10.3389/fdata.2019.00050 (PMC7931954; doi:10.3389/fdata.2019.00050)
Supplement: Supplementary file 2 [file Data_Sheet_1.PDF]

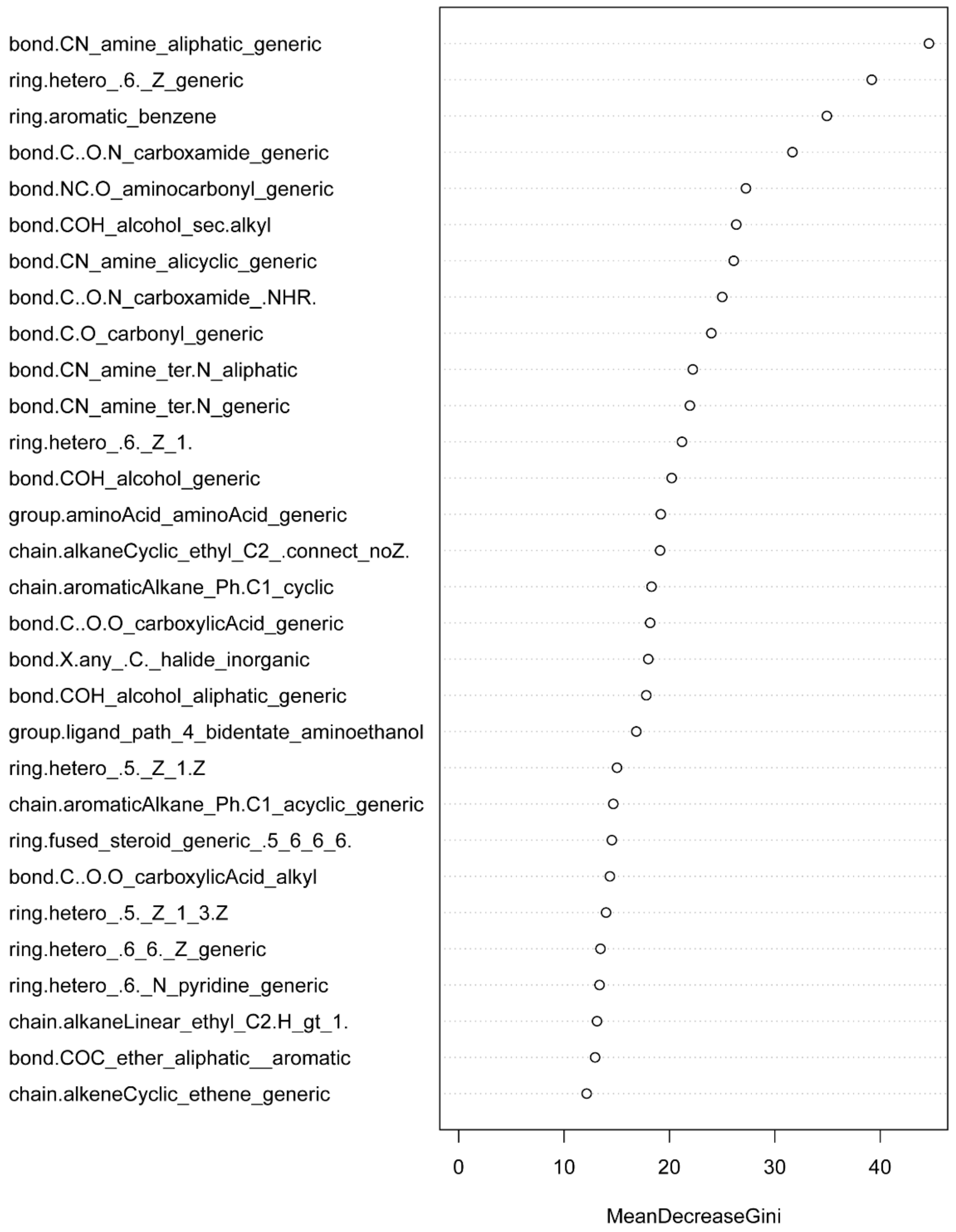

Supplementary Figure 1: Variable Importance Plot for chemical structure features generated by Random Forest method. The Mean Decrease in Gini, a measure of variable importance for estimating a target variable, was calculated for the top 30 chemical structures critical for compound classification. The most important chemical structure features are located at the top of the plot and have the largest Mean Decrease in Gini values.

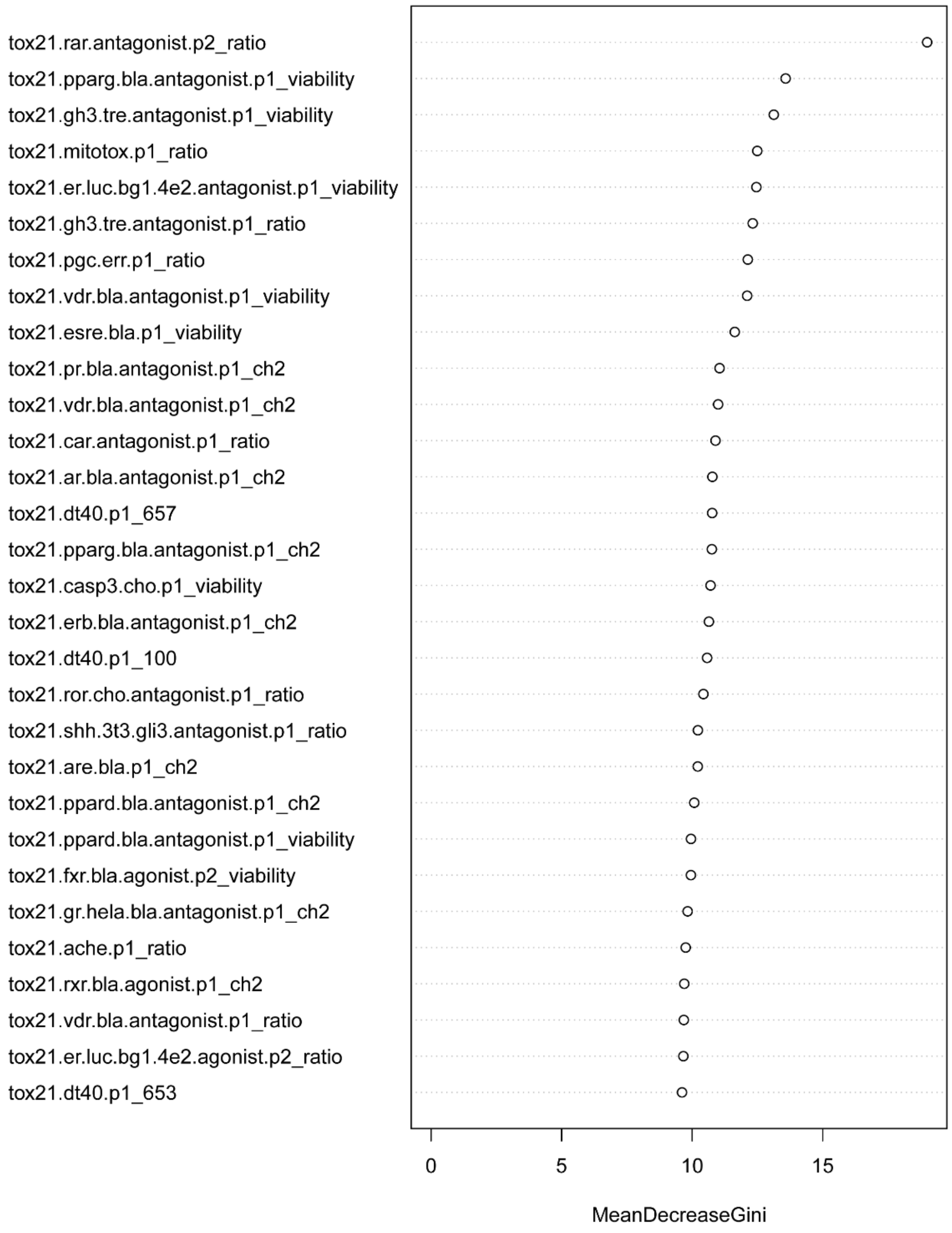

Supplementary Figure 2: Variable Importance Plot for Tox21 assays generated by Random Forest method. The top 30 Tox21 assays are shown, with the most important assays located at the top of the plot with the largest Mean Decrease in Gini values.
